# Support in Daily Living for Young Adults with Neurodevelopmental Conditions in Sweden: A Qualitative Description of Current Practice

**DOI:** 10.1007/s10803-023-06014-6

**Published:** 2023-05-23

**Authors:** Maria Löthberg, Tatja Hirvikoski, Sonya Girdler, Sven Bölte, Ulf Jonsson

**Affiliations:** 1 Center of Neurodevelopmental Disorders (KIND), Centre for Psychiatry Research, Department of Women’s and Children’s Health, Karolinska Institutet & Stockholm Health Care Services, Region Stockholm, Stockholm, Sweden; 2 Habilitation and Health, Region Stockholm, Stockholm, Sweden; 3 Child and Adolescent Psychiatry, Stockholm Health Care Services, Region Stockholm, Stockholm, Sweden; 4 Curtin Autism Research Group, Curtin School of Allied Health, Curtin University, Perth, Australia; 5 Department of Medical Sciences, Child and Adolescent Psychiatry, Uppsala University, Uppsala, Sweden

**Keywords:** ADHD, Autism, Content analysis, Daily living, Social services, Transition

## Abstract

In Sweden, people living independently and requiring daily living support can access ‘housing support’, a form of practical, educational, and social support provided by the municipalities. About two-thirds of those receiving this support have neurodevelopmental conditions, primarily autism or ADHD. Many are young adults in the process of adapting to new roles and expectations in different life domains, including education, work, and accommodation. This study aimed to provide a qualitative description of support workers’ views on current practice in housing support for young adults (aged 18 to 29) with neurodevelopmental conditions. Semi-structured telephone interviews were conducted with 34 housing support workers across 19 Swedish regions. An inductive qualitative content analysis approach was used. The interviews depicted a complex service, subject to *organizational* aspects (roles, responsibilities, availability, and allocation), the joint effort of *key players* (young adults, relatives, and support workers), and practical aspects of *service provision* (finding common ground for the work, and delivery of support). Some elements of the service were poorly designed for the target group. The support workers expressed a need for more knowledge about neurodevelopmental conditions, but also described new insights related to remote delivery of support. The results raise important questions about how housing support should be organized and delivered to strike the right balance between support and autonomy, meet specific needs, and ensure equal services across municipalities. Future research should adopt multiple perspectives and approaches, to help translate best practice and available evidence into a flexible and sustainable service.

The transition from adolescence to adulthood is a period in life characterized by increasing independence and exploration of adult roles and relationships, gradually leading to enduring decisions and structure in everyday life (Arnett, 2000). During this process, young people are increasingly expected to navigate life themselves, and adapt to societal roles and expectations, eventually achieving milestones such as leaving home, completing their education, or finding a job (Grant & Potenza, 2009). Successfully achieving these milestones is challenging, requiring greater personal responsibility, complex decision-making, and organizational skills (Arnett & Padilla-Walker, 2015; Ayotte et al., 2020).

This process is often perceived as particularly difficult for young adults with neurodevelopmental conditions (NDCs) (Di Lorenzo et al., 2021; Howlin, 2021), including autism and attention-deficit/hyperactivity disorder (ADHD) (American Psychiatric Association, 2013; World Health Organization, 2019). Functional challenges in several domains (e.g., decision-making, problem-solving, daily routines, housework, distributing energy and drive on different objectives, and managing basic interpersonal interactions) (Bölte et al., 2018, 2019) and insufficient environmental accommodations (Mahdi et al., 2017, 2018) can complicate the daily lives of young people with NDCs. The cumulative impact of impairing individual and contextual factors is evident in the adverse social and health-related outcomes (Anderson et al., 2018; Fuentes et al., 2020; Hirvikoski et al., 2016; Ljung et al., 2014) and poor quality of life (Jonsson et al., 2017; Steinhausen et al., 2016; Thorell et al., 2019) experienced by this group.

Support and environmental accommodations are often needed in several life domains, including personal finances, housing, education, work, health, and relationships (e.g., Elster et al., 2020; Sansosti, Cimera, et al., 2017). Although research suggests beneficial effects of interventions focusing on self-determination (Nadig et al., 2018), social skills (Chancel et al., 2022), managing barriers within educational or work environments (Anderson & Butt, 2018; Lyhne et al., 2021), transitioning between school levels (Devoe et al., 2022), from school to work (Baker-Ericzen et al., 2022) or between care providers (Eke et al., 2020), these supports are often fragmented and context-bound (Bottema-Beutel et al., 2022). In addition, the overlapping nature of conditions such as autism and ADHD (Antshel et al., 2019), and the presence of co-occurring mental health conditions such as anxiety, depression, and sleep disorders, can further complicate the young adult’s life situation (Abecassis et al., 2017; Howlin, 2021; Martini et al., 2022). For this reason, promoting optimal outcomes for adults with NDCs may require joint efforts from health care and social services.

Sweden, like other Scandinavian countries, prioritizes social and public spending on incapacity (OECD, 2022) and publicly funded welfare. Welfare services in Sweden are managed by two legislated public organizations: 21 regions, each governing regional health care service; and 290 municipalities, responsible for social services. According to the Swedish Social Service Act (SoL 2001: 453), people living independently and requiring support in daily living can access ‘housing support’, a form of practical, educational, and social support provided by the municipalities. While national legislation mandates the right to access housing support based on impairments and support needs, the decision to grant support and planning for its delivery is decentralized to the municipalities. Based on an individual’s profile and needs (usually supported by a psychiatric assessment), eligibility for this support is assessed by a social services care administrator in the municipality of residence and is terminated if a person moves to another municipality. Requesting support is self-determined and voluntary, and there is no limit to the number of times an individual can apply. The aim of housing support is to enhance an individual’s independence and ability to manage their everyday life, both within the home and community (Swedish National Board of Health and Welfare, 2022).

The number of Swedes accessing housing support has increased by more than 200% from 2009 to 2019 (Swedish National Board of Health and Welfare, 2020) to the extent that it is now the most frequent support service accessed by those aged 18 to 34 years (Swedish National Board of Health and Welfare, 2021b). This increase has partly been attributed to a national deterioration in the mental health of Swedish young adults (Swedish National Board of Health and Welfare, 2020). Given approximately two-thirds of those receiving housing support are reported to have an NDC (Swedish National Board of Health and Welfare, 2019), there is a need to consider the specific support needs of this group and whether support workers possess the qualifications to adequately support young adults with NDCs. While there are no mandated educational requirements for housing support workers, they generally have educational backgrounds in nursing or social care, e.g., assistant nurse (Swedish National Board of Health and Welfare, 2022). More recently those with qualifications in social work or behavioral science (Swedish National Board of Health and Welfare, 2022) are being employed, indicating that these fields align with the requirements of these positions. Currently, support workers are predominantly female (Statistics Sweden, 2022).

With the goal of delivering person-centered support services municipalities are encouraged to actively involve their clients in planning and implementing support, minimizing the number of workers involved to foster trusting working relationships, and maintain continuity (Swedish National Board of Health and Welfare, 2022). While housing support is available nationally in Sweden there are no national guidelines detailing how these services should be delivered. However, some key components have been suggested, including the content of the support in terms of activities of daily living, the location where the support is provided (in the individual’s home or in the community), and the relationship between the individual and the support worker (Swedish National Board of Health and Welfare, 2010a). Similarly, a qualitative study focusing on the experiences of young adults (aged 20 to 35) underscored the importance of the dialogue, the mentor-like relationship, as well as whether the young adult was living with parents or independently (Andersson & Gustafsson, 2014).

Municipalities and service providers are largely free to design their own model of service. However, the service should be underpinned by the fundamental principles of individuality, predictability, continuity, and equality (Swedish National Board of Health and Welfare, 2022). Achieving this, while simultaneously aligning the service with the needs of a specific target group (Renty & Roeyers, 2006; Swedish National Board of Health and Welfare, 2010b), is a complex task. Adding further to the complexity, the COVID-19 pandemic disrupted the delivery of community services in Sweden (Fridell et al., 2022; The Public Health Agency of Sweden, 2021) and globally (World Health Organization, 2020). This raises important questions about how housing support is provided for young adults with NDCs. In adapting services towards this specific target group, service providers must consider interpersonal aspects, environmental accommodations, different models of service delivery, and intersectoral collaboration. In doing so, they must draw knowledge from the fields of psychiatry, social work, psychology, education, and occupational therapy. Currently, it is unclear how municipalities deal with this complexity.

To our knowledge, no research to date has described current practice in providing housing support to young adults with NDCs. Understanding the scope of housing support for young adults with NDCs across Swedish municipalities is crucial to guide policy and practices toward the more efficient provision of support and identify areas for improvement. Thus, this study aimed to provide a qualitative description of current practice in housing support for young adults (age 18 to 29) with an NDC based on the views and experiences of housing support workers.

## Methods

### Design

This was a qualitative description of support workers’ lived experience of delivering housing support, entailing low-inference interpretation (Sandelowski, 2000). A constructivism/interpretivism paradigm was used, recognizing the subjective nature of the participant’s experiences and the relevance of the researchers’ values and beliefs (Ryan, 2018). Throughout the process, we have used the Checklist for Researchers Attempting to Improve the Trustworthiness of a Content Analysis Study by Elo et al. (2014).

### Participants and Recruitment

To gain access to a wide diversity of experiences (Elo et al., 2014), we used a purposive sampling framework intending to capture the range of approaches and experiences arising from variations in population density and/or geographical location. The aim was to recruit participants from municipalities representing each of Sweden’s 21 regions. To ensure that municipalities of different population sizes were represented, municipalities within different population ranges (< 20 000; 20 000 to 100 000; and > 100 000) were approached. The aim was to recruit seven municipalities within each range.

Municipal housing support providers in 56 municipalities (out of 290), dispersed over all 21 regions and with populations ranging from 5400 to 970 000, were contacted and provided with verbal and written information about the study during the period October 2020 to May 2021. The study was introduced to staff by their line manager. Support workers with lived experience of delivering housing support to the target group contacted the project coordinator who provided written information and an opportunity for questions prior to participants providing their consent to participate. A mutually convenient time to conduct the interview was then scheduled.

A total of 34 support workers (26 females, 8 males) shared their professional experience (median 7 years work experience; range 2 to 30 years) from delivering housing support to the target group. Fifteen of the participants had education in health care, mainly nursing, while the remaining reported having received education in psychiatry, behavioral science, social work, or pedagogy.

Participants represented 22 municipalities (9 municipalities with populations over 100 000; 7 municipalities with populations between 20 000 to 100 000; and 6 municipalities with populations less than 20 000) from 19 different regions across Sweden. In some municipalities, the providing of housing support was organised with a team providing support to specifically service users in the target group of young adults with NDC. This was not the case in other municipalities, where teams provide housing support to all those who are granted this service, ages 18 to 65, with a variety of impairments, including young adults NDCs.

### Interviews

Telephone interviews with housing support workers were conducted in November and December 2020 and February to March and May 2021, either individually (n = 14) or in groups of two to four support workers (n = 8), lasting between 30 and 60 min. To support participants in preparing for the interviews, an interview guide was sent in advance. Participants were free to discuss the questions in the team before the interview, to be able to represent the team’s discussion and collective views during their interviews. In 16 of the 22 participating municipalities, the participants discussed the questions with parts of or the full team.

The open-ended interview guide comprised seven questions (Table 1) and aimed to direct the interviews toward participants’ experiences and views of providing housing support specifically to young adults (aged 18 to 29) with NDCs. Being well known and common conditions, ADHD and autism were used as examples to guide participants to focus on the intended target group of service users during the interview.

**Table 1:** Interview guide

Q1. How long have you worked in housing support?
Q2. What is the purpose of housing support for young adults with autism and/or ADHD?
Q3*. What are the needs of young adults with autism and/or ADHD that are met by housing support?
Q4*. Describe the ways you work with these young adults in providing housing support.
Q5*. How do you decide what to work on and how the support should be delivered?
Q6*. What barriers or factors influence the supports you provide to these young adults?
Q7*. What do you think could improve the support services provided to young adults with autism and/or ADHD?

Note. *Q3-Q7 included a follow-up question regarding the impact of the COVID-19 pandemic

Follow-up questions were utilized by the interviewer to clarify and further explore the participants’ responses. Interviews were conducted by the first author (ML), a doctoral student with a master’s degree in pedagogy and seven years of work experience in a specialist group providing housing support to young adults with NDC within the social service organization of the municipality of Uppsala. The municipality of Uppsala was not invited to participate in the study to ensure the impartiality of the interviewer. Clean verbatim transcriptions of all audio recordings of the telephone interviews were performed by an external company, generating 300 pages of written text.

### Data Analysis

An inductive qualitative content analysis approach was used in analysing the data (Graneheim et al., 2017; Graneheim & Lundman, 2004). Before commencing data analysis, interview transcripts were compared with audio recordings by ML, ensuring the accuracy of the transcription. To familiarize themselves with the interviews ML and UJ read all transcripts, independently identifying meaning units (sentences or paragraphs) in three interviews, achieving consensus via discussion. Subsequently, ML analysed the remaining interviews, labeled meaning units with a code that maintained the manifest content of the unit. When necessary, ML discussed her approach with UJ, to ensure intersubjectivity and full exploration of the transcripts. Across the interviews, codes were then grouped into subcategories, categories, and later main categories, progressing from information and manifest content to a higher degree of interpretation and abstraction (example shown in Table 2). This categorization process, performed collaboratively by ML and UJ, required working iteratively with the material, moving between interview transcripts and analysis, comparing, and reviewing their content and composition (Elo et al., 2014). The categories were subsequently refined after discussions within the authorship team. The software NVivo 12 (QSR & Ltd., 2020) assisted with organizing data and supporting analysis. Interviews were numbered consecutively (P-01 to P-22). In reporting the results, relevant quotations from several interviews are used to illustrate categories.

**Table 2:** Example of the analysis

Meaningful unit	Code	Subcategory	Category	Main category
’We try to match who goes to whom and who is best suited. We try as best we can, but it’s not always possible…’(P-12)	Matching	Staffing	Availability and allocation	Organization
’… maybe mum or dad thinks that the youth should go out for a walk, but the youth himself doesn’t want to. (…) Then the parents feel that we should do as they say. (…) Then I usually explain that “your son or daughter is actually of age, we have no right to decide what he or she should do”. If he or she doesn’t want to go out, we can’t force him or her. This can actually cause some problems.’ (P-15)	Parents want to decide	Asset and discordance	Relatives	Key players
’… they [young adults] may not be aware of what needs to be done. It still makes them feel bad, and maybe they don’t see that it is cluttered or whatever. Somehow, we can provide an external perspective and help them see their home differently.’ (P-02)	Help to clarify needs	Methods and strategies	Delivery	Service provision

## Results

Data analysis resulted in three main categories: Organization; Key players, and Service provision. Table 3 presents an overview of the main categories, categories, and subcategories.

**Table 3:** Overview of subcategories, categories, and main categories

Subcategory	Category	Main Category
Assessment and support plans Role ambiguity	Roles and responsibilities	Organization
Staffing Time management	Availability and allocation	
Support needs Motivation	Young adults	Key players
Asset and discordance Living with others	Relatives	
Diverse teams Dedication and frustration	Support workers	
Working-alliance Involving the network	Finding common ground	Service provision
External challenges Methods and strategies	Delivery	

The main categories and their comprising elements are described below, including pertinent quotations from interviewees.

### Organization

Participants raised several issues relating to the role of housing support in the organizational structure of municipalities and in allocating resources.

#### Roles and Responsibilities

*Assessment and support plans.* Participants highlighted the importance of assessment and support plans in defining the services they provided. Social services arrange housing support, where care administrators assess clients’ needs and formulate support plans, passing these on to the service teams that deliver support. While participants highlighted the importance of these assessments in determining a young adult’s support needs, they pointed out that difficulties in initially identifying support needs could lead to inaccurate support plans. Support plans were key in enabling participants to track their client’s progress over time, guiding the support they delivered, and assisting them in formulating achievable goals with their clients. Several participants noted that at times goals formulated by the care administrator in the initial plan were too ambitious, requiring work in reframing and prioritizing goals.

‘… it is often the case that we have to start with sorting out all the contacts with different authorities. Because that is what is most urgent, you know. You must let go of this idea that it should be clean and tidy at home.’ (P-13).

Furthermore, municipalities varied in how they enacted support plans. In some municipalities support plans were strictly followed, in others support plans were seen as a starting point from which to map and explore a client’s needs collaboratively, involving a feedback loop with the administrator.

The provision of housing support was monitored by care administrators. However, the frequency of this monitoring varied, and was often constrained by available time. When young adults no longer require support, being satisfied with achieving their goals, housing support ceases.

‘We are present when we are needed … and if we are no longer needed one day - great!’ (P-02).

Although several participants reported instances where this happened, others noted that this outcome was quite unusual. One participant noted that a clearer support strategy can be achieved if the plan also specified when the support should be terminated.

‘What’s the plan? What are we thinking? How long will it take before we can terminate the support? We could do much better in that respect.’ (P-14).

*Role ambiguity.* Participants described that the role of support workers was sometimes perceived differently by managers, care administrators, and support teams within the organization, expressing that at times there was a lack of clarity regarding the types of support they should provide, the required skills and knowledge profile, and their role in determining when support needs had been met. This lack of consensus negatively impacted in-house cooperation and support workers’ understanding of their role and its requirements. Several participants highlighted the paradox that while accepting housing support was mandated as voluntary, at times accepting support was a condition of obtaining housing:

‘[A client] has been granted a transitional contract for an apartment, where the condition for obtaining this apartment has been to accept housing support. But this is a voluntary support service, and it becomes a conflict… we’ve assured and told the service user that, “You can say no to the support if you want to, that’s okay”… and this makes it difficult for the individual, I would say.’ (P-04).

Multiple participants noted that support workers at times played an important role in directing clients to other services, such as health or employment services. Regarding the evaluation of support needs initiated by the care administrator, guiding the young adults towards more extensive support was described as a delicate task.

‘… We are used as a tool to… it is also important to show when [housing support] does not work, that our resources are not enough … because if you cover up all these needs that many service users have, it does not show, and then “well, everything works all right”, yes but we do so much more than we should in this case.’ (P-13).

Moreover, participants described the negative impact of the COVID-19 pandemic on the young adults’ social networks and community mobility, noting that in their role they often filled the gap of lost social contacts.

#### Availability and Allocation

*Staffing.* The process of allocating support plans to workers varied across municipalities. Some attempted to match support workers with service users, while others allocated plans based on support workers’ availability. To provide continuity of service and promote productive working relationships some municipalities aimed to limit the number of support workers involved with each young adult to one or two. However, several participants raised that this practice made it difficult to provide support when a designated support worker was unavailable, encouraging the young adults to interact with several support workers.

‘There are some service users who want fewer… staff and then we try to be responsive to their request, but we cannot make promises … if none of the individual´s support workers are working that day, or at that time… then they have to work with someone new… and then it is up to the service user if they want to expand their network and meet more people, or if they choose to cancel the support that day.’ (P-04).

Numerous participants noted that the workforce shortages resulting from the COVID-19 pandemic were problematic, disrupting the continuity and delivery of services.

*Time management.* The hours of available support varied across municipalities, with some offering services seven days a week and into the evenings, and others providing services only during office hours on weekdays. While some municipalities offered regular times for support meetings, others arranged appointments on a week-to-week basis. Several participants noted that it was often difficult to align a young adult’s wishes with the availability of staff:

‘We can’t promise a fixed day and time every week to any service user since that is not how the staff are scheduled.’ (P-03).

Some participants noted that limited access to cars negatively impacted their ability to provide services and respond to young adults’ needs:

‘You probably would have needed half an hour or more to get further and… it [the schedule] is a vulnerable structure. And the barrier can be related to all the logistics and everything. … That we might have to carpool with someone and… I have to be in the parking lot at a certain time.’ (P-13).

Several participants also expressed that they wanted more time to reflect on their work and to complete administrative tasks. Participants noted that during the COVID-19 pandemic, there was, at times, greater flexibility in scheduling appointments because of cancellations, allowing for additional support meetings or administration time.

### Key Players

Participants perceived that while the model of delivering housing support was significantly influenced by the young adults and their needs, the attitudes and involvement of relatives and support workers were also decisive.

#### Young Adults

*Support needs.* Most participants highlighted that the central aim of housing support for young adults with NDC was to enable them to live in their communities as independently as possible. In meeting this aim participants worked with their clients in structuring meaningful daily routines in domains such as home life, personal finances, and occupation, reducing their client’s social isolation and facilitating their connection with government support and healthcare providers.

‘It is very practically oriented, what we do. To practice cleaning or washing to become more independent. Focus on planning, using a calendar, planning your everyday life. It can also be about inclusion in society…. looking for jobs, attending meetings, writing support …’ (P-19).

Participants described the support needs as changing over time, with a decreasing need for guidance in basic daily living activities as the service users approach their thirties.

‘… it’s mainly towards those who are in this target group who are a little bit younger, those who are a little bit older you don’t have to guide them from morning to night, but it’s more with those who are a little bit younger, that you have to structure their whole life for them to get started. From eating breakfast to showering to going to work. When to do the laundry? When to clean? When to shop and cook and… so it’s quite extensive I think with those in this target group.’ (P-07).

‘… we increasingly get younger people, younger people receiving housing support, and also people who never go out, to get them to move out, into a place of their own and start living.’ (P-05).

Participants highlighted that living independently provides an opportunity for young adults to focus on building autonomy and self-reliance, but also noted that the responsibilities of running their own households were demanding and at times stressful (e.g., difficulties establishing and maintaining routines and structure, carrying out housework, time management and problem-solving).

According to the participants, the service could be impacted by young adults forgetting ‘about time and space’, getting stuck at the computer, double booking meetings, or choosing to see friends instead of the support worker.

‘This target group can sometimes be very difficult to reach. I mean, they are young, they want a lot of fun, and they are impulse-driven.’ (P-10).

The young adults often needed support in specific situations, to not lose their train of thought, manage impulsivity, or initiate activity. Typical situations were shopping, transportation, and contact with authorities and health care providers. The latter included face-to-face meetings, phone calls, and handling of documents. Many participants also describe the young adults’ need for reminders to manage, for example, medicines, bills, appointments, and school/work. Young adults often failed to see the consequences of neglecting such responsibilities.

‘They’re high functioning, skilled, but they can’t really see consequences, if I do this, this is what happens. Many times, it [understanding consequences] is not there at all.’ (P-01).

Moreover, participants described that the young adults’ support needs varied as a result of fluctuations in their health. Unstable mental health and substance use were described as common reasons for cancelling appointments or terminating support plans.

Participants noted that their clients with NDC accessing housing support services were heterogeneous regarding their age, level of functioning, family situation, accommodation, and insight into their abilities and disabilities. Collectively, these factors impacted clients’ support needs and the services they require.

*Motivation.* Nearly all participants noted the importance of a young adult’s willingness to work collaboratively and readiness for change as key to providing successful support.

‘The individual must actively take part and do something on their own. Accept. Try. … You must be willing to do something. In any case, accept the housing support. You know, answer the phone, or open the door.’ (P-03).

Participants highlighted that the young adults should be ‘on board’ when the application for housing support is made, noting that unmotivated clients made their work difficult, often resulting in canceled meetings and premature cessation of support.

‘…those who apply might not want housing support. We’ve noticed that. Maybe it’s a mom or a dad, or the psychologist, who wants to… It’s quite often the case, especially when they’re younger.’ (P-08).

Conversely, sometimes the support provides the young adults with a social context and security, which can make the young adults reluctant to end their support.

#### Relatives

*Asset and discordance.* While participants described the parents of young adults as ‘an asset’ and ‘collaborators’, sharing information about their children and being there as a ‘backup’, at times parents were perceived as non-supportive, ‘doing too much’ or attempting to micromanage the housing support.

‘If it’s about cleaning up… It may be that the relatives have already cleaned up … the work will be more difficult then. The person does not see the consequences of… not cleaning up, because they don’t have to take that responsibility.’ (P-04).

Participants described that the presence of parents at times challenged their ability to provide a confidential service to their clients or distract the young adults during meetings. Further, while some housing support teams provided family support as part of their service, other teams directed relatives to other services.

*Living with others.* Participants expressed that at times working within a young adult’s home environment was difficult. It was discussed by participants that housing support workers sometimes played a role in building the skills of a young person (e.g., housework, finding a vocation, and managing contacts with authorities) prior to moving out of their parental home. However, living in a parental home could limit opportunities for practicing skills such as cleaning or cooking (e.g., due to routines already in place in the family). It was also acknowledged that at times the transition to independent living was abrupt, with no time to focus on building their skills.

‘Maybe they haven’t done much at home with their parents [in preparing for the move out of home] … and then they find themselves on their own with everything. Yes, we increasingly see this.’ (P-16).

The need for semi-independent supported accommodation settings, providing graded levels of support to young people with NDC working towards moving out of home was also raised.

When the young adults lived with others such as with parents, partners, or children, participants attempted to schedule appointments to coincide with times when others were not at home, allowing the young adult to focus on the tasks at hand.

Participants noted that major changes in young adults living situations, such as finding a partner or having children, were often a catalyst for terminating housing support with remaining needs commonly met by relatives.

#### Support Workers

*Diverse teams.* Several participants highlighted the key role of the service team, seeing diversity in terms of age and experience as an asset. The wider service team supported participants in providing advice and acting as a ‘sounding board’ when solving problems about contacting a client or discussing their concerns.

‘You need to exchange ideas. You need to find new strategies and that’s what we do a lot in the team.’ (P-16).

Several participants pointed out that they themselves and their experiences are a tool in the work, and several of the participants would like to have more access to external guidance or supervision.

*Dedication and frustration.* Many participants described being highly committed to their work, attempting to flexibly meet their client’s needs, often making themselves available outside of working hours, and at times ‘bending the rules’.

‘When we go home at five o’clock, we sometimes bring the phone home and take an extra call later in the evening, because of the state the individual is in. That alone will make some persons [young adults] feel safer.’ (P-09).

Participants reported that at times in ‘doing their job’ they challenged their client’s autonomy and self-determination.

‘It can also be anxiety-provoking, I think, for the client to get a text message from us. We’re pushing on because we want to do our job. We want to get going. If they’re not motivated, then they will have to come up with a reason as to why they don’t want us to come.’ (P-07).

While participants highlighted the inherent potential in young adults, they were frustrated when things did not go to plan. Clients at times prioritized tasks differently than the support workers. Missed appointments were frustrating to participants, particularly when they had to travel large distances to visit a client.

‘…you have to talk about it the next time you get there, that “if you don’t want me to come then you must let me know so I don’t have to travel. It takes 2.5 hours to get here.”’ (P-21).

‘… I had arranged to get there at 11 a.m. and he’s asleep. Then he gets, then he’s so ashamed when I ring on the doorbell. “No, I can’t open. This is so damn embarrassing”.’ (P-17).

### Service Provision

Throughout their interviews participants reiterated that in delivering housing support to young adults with NDC, they drew on their professional and interpersonal skills, in building a working alliance with the young adults, adapting the support to the young adults’ current needs, and involving the network of service providers.

#### Finding Common Ground

*Working alliance*. Building a relationship with their clients founded on mutual respect and shared understanding was commonly noted by participants as the most important factor underpinning their work. Participants highlighted the importance of being adaptable and flexible in aligning their approach with the needs of each young adult, of being responsive, curious, and humble, and respecting an individual’s own pace in building a working alliance.

‘Because if you can’t get people to trust you, they won’t accept the support. (…) So that’s it, that’s the most important thing. And it’s absolutely the first thing you do in building an alliance.’ (P-03).

Participants mentioned the client’s insecurity to let the support worker into their home, noting that they often began by meeting with clients on more neutral ground in community settings outside of their homes.

‘It’s a little scary just to let us in [to the home] … you know, will there be a lot of people at my place?’ (P-01).

Participants noted that their role required patience and persistence over time, and a commitment to building rapport with their clients by keeping in touch through multiple communication channels (e.g., telephone, text messages, mail, letters, and network), and not giving up easily.

‘Sometimes you have to be insistent and just keep going. With one girl, I don’t know how many cancellations I had. But I didn’t give up … But it took so long before she… dared to let me in’ (P-08).

A trusting relationship enabled constructive dialogue between participants and their clients, making it possible for participants to challenge the young adults´ attitudes and beliefs.

Participants described the delicate balance between ‘getting things done’ and empowering their clients to ‘do things for themselves’. Support workers saw themselves as ‘acting as a sounding board’ without being domineering, and over time as enabling their client’s independence.

‘You are not allowed to make the decisions for somebody, but at the same time you must give advice with some authority so that it is worth listening to, and stress that things need to be done.’ (P-18).

Most participants expressed that being sensitive to the young adults´ need for self-determination and respecting and accepting their choices was important to their work.

*Involving the network.* Participants stressed the importance of clarifying expectations, not only for the young adults but also for their relatives and other professionals involved.

‘We’re not just someone who is fun to talk to, we’re actually there to do some work.’ (P-03).

Providing ‘good support’ was dependent on connecting with the young adult´s wider support network, and collaborating closely with the other professionals involved:

‘… and make it clear to this person [young adult] what everyone is doing. Because I often feel that they don’t understand, they have so many people around them and don’t really know who’s doing what.’ (P-15).

Participants described that providing ‘good service’ depended on input from the whole network and that at times, a lack of effort from other service providers posed a barrier to achieving a good outcome for the client.

‘…our work has come to a halt with this client now, nothing is happening, because of the situation with the Social Insurance Agency, the Doctor, it does not work.’ (P-05).

Overall, participants highlighted that more effective collaboration between all stakeholders including relatives, care administrators, municipal activities, authorities, employers, and social services and health care services could significantly improve the support services to young adults with NDC.

#### Delivery

*External challenges.* Several participants describe contextual/environmental aspects which impacted on and sometimes complicated the provision of service. According to several participants, for example, young adults could feel exhausted after completing their work or education commitments, making it challenging to organize support meetings. Also, the young adult’s limited financial resources were a key barrier restricting their participation in social or sporting activities with their support worker, and in accessing care and support when required to pay for transport or other associated costs (e.g., lunch bag for long trips to health care visits).

Notably, the onset of the COVID-19 pandemic significantly impacted the way in which participants delivered their support to their clients, including limiting face-to-face meetings, shortening meetings, holding meetings outdoors, and shifting to digital solutions such as phone calls and video meetings. The rapid change negatively impacted their contact with individuals, collaboration partners, and colleagues. Not being able to provide support in the individual’s homes or practice activities in society were highlighted by many interviewees as the most difficult to manage.

Sweden’s public health response to the COVID-19 pandemic, including social distancing and recommendations to work from home or study remotely, required self-discipline and flexible working practices by the young adults, which in turn affected the focus of the housing support services.

‘But you try your best, maybe do some extra schedules [for the young adult] and help with a place to sit when they [the young adult] do their work. Perhaps arrange a small work corner and try to help. It has become more difficult, I have to say.’ (P-20).

*Methods and strategies.* Rather than drawing on a specific theory or method, most participants adopted an eclectic approach, adopting a solution-focused approach, or problem-solving strategies. Several participants adopted a more structured model such as case management. Others described aligning their approach with each client’s needs, often employing motivational interviewing which was seen as a key aspect of housing support. Other approaches mentioned included social skills training and planning tools. The importance of some practical aspects was also stressed. Participants described aligning their model of service delivery with each client’s needs, providing their services in-situ as a way of modelling or teaching processes, or prompting or shaping daily activities remotely over the telephone. Several participants noted that the opportunity to converse with clients while traveling together in the car was important to their work.

‘… in this working group, we have always used the car as an important tool. It’s been so good. You can have really good conversations in the car.’ (P-10).

Participants stressed that delivering housing support was a collaborative effort, requiring negotiations with clients in establishing their short and long-term goals. They described working with their clients to explore their needs and to identify the most appropriate support.

“You have to look into these little details. To ask further questions all the time. ‘What do you mean? How do you mean? Why is this not possible for you?” (P-01).

Being flexible and responsive was also key in enabling participants to appropriately respond to the fluctuating health and needs of the young adults they supported (e.g., being available via telephone).

‘We are very compliant. I mean with the well-being of our clients. So, it shifts quite often and then we adapt and give more support when needed and step back when we see that it is possible.’ (P-10).

All the participants described how the COVID-19 pandemic entailed more frequent use of digital tools and several participants mentioned that they themselves had to learn things that were already known by many young service users.

Some of the support workers’ ideas about their work had been challenged. Some described that some young adults made even greater progress toward independence during the pandemic.

‘We’ve had some [service users] that we’ve had a hard time getting started, and when we’ve changed to, instead of having two face-to-face meetings per week, instead have 15 minutes on the phone every day, suddenly it works!’ (P-14).

Several participants argued that these positive experiences and new insights should be utilized within housing support.

‘We’ve talked about this, that we see other things and we have to cherish these and not go back to old ways. Because then we would counteract their [the young adult’s] progress now that they got started.’ (P-01).

Participants noted that their ability to support their clients could be improved by greater access to digital tools, and further education about NDC and those factors key to delivering effective support.

## Discussion

This study explored Swedish housing support workers’ views on providing support in daily living to young adults with NDCs. No detailed account of the nuts and bolts of this Swedish welfare service for this target group has previously been available. The findings highlighted the complexity and heterogeneity of current practice, which seems to vary considerably depending on the young adult’s motivation and current situation, the support worker’s skills and general approach, and organizational differences across municipalities. The results raise important questions about how the support should be organized and delivered to strike the right balance between support and autonomy, meet the specific needs of this target group, and ensure equality across municipalities.

The main objective of housing support, as expressed by the interviewees, is to facilitate a gradual increase in autonomy and independence rather than to “provide a service”. Consequently, the support worker faces the challenge of finding a way of being supportive, but not governing, and incorporating the young adult’s objectives and self-determination as integral parts of the support provision. Drawing from self-determination theory (Ryan & Deci, 2000), seeking to enhance young adults’ competence and autonomy to increase their intrinsic motivation to take active participation to achieve individual goals seems crucial for sustainable change. The service focuses not only on instrumental support in daily life, but also on strategies to facilitate social engagement and personal development, something that the interviewees described in terms of mentoring. Examples provided by the support workers included the importance of building a trusting relationship, taking the role of a sounding board for the young adults, and replacing other social contacts during the pandemic. A change from family-based support to more community-based support can be seen as a step toward increased independence (Andersson & Gustafsson, 2014). The friction sometimes experienced by the support workers in their contact with relatives might be seen as a result of this shift, including parents taking over the tasks from the young adults or attempting to micromanage the housing support. Overall, the importance of good collaboration with relatives and the broader network around the young adults was emphasized.

Importantly, steps towards increased autonomy and independence (e.g., getting a job) can lead to stress and increased demands (Ahlström & Wentz, 2014; Eklund et al., 2017), which in turn can generate new support needs. Some organizational aspects (e.g., that support only is available during business hours or lack of coordination with other stakeholders) seem to constitute virtual barriers for young adults who start an education or enter the job market to still get the support they need. Importantly, effective coordination between stakeholders (e.g., healthcare services, social services, authorities, and employers) might be crucial to enable such steps. A range of barriers to service integration in complex and fragmented service systems have been reported (Trane et al., 2022). From the perspective of the support workers interviewed in the present study, knowing who to contact is an important prerequisite to improved collaboration. Similarly, there is a risk that a decrease or termination of housing support as young adults gradually become more independent can lead to social isolation (Brolin et al., 2015; Sandhu et al., 2017), and reduced quality of life (Brolin, 2016). To counteract this, there is a need for complementary support tailored for specific contexts, like peer mentoring in education and work (Lindsay et al., 2016; Nguyen et al., 2020; Thompson et al., 2020). A gradual and well-planned withdrawal of support over an extended period of time during the maintenance phase may be advisable, particularly for young service users. It is also important to note that some support needs can be life-long, and might even become more pronounced over time, in which case a certain level of support will be required across the lifespan.

At times, current practice appeared to be guided primarily by organizational needs (e.g., staffing, working hours, and means of transport), without fully considering the specific needs of the target group. For instance, the erratic nature of current practice in some municipalities (e.g., flexible schedules and contacts with multiple support workers) stands in contrast to the need for predictability and continuity often experienced by people with NDCs (Andersson & Gustafsson, 2014; Brolin 2016; Brunt & Tibblin, 2016; Sosnowy et al., 2018). This is likely to have a negative impact on motivation, even discourage some young adults from participating.

### Future Directions

There is a body of research emphasizing the need for more holistic support for young people with NDCs (Baric et al., 2016; Fuentes et al., 2020; Sansosti, Merchant, et al., 2017; Song et al., 2022; Sosnowy et al., 2018). While housing support has the potential to deliver a holistic support service to young adults with NDCs, particularly given there are no specified limits in terms of hours or target age, this study suggests that in realizing this goal several issues should be addressed. In doing so, a combination of research perspectives and methods will be needed, in line with the current framework for developing and evaluating complex interventions (Skivington et al., 2021).

To begin with, the use of more standardized elements is a possible way to improve both quality and equity in service provision. For example, increased use of the International Classification of Functioning, Disability and Health (ICF) (World Health Organization, 2001) could facilitate a more systematic approach, focused on individual needs (Bölte et al., 2021). Similarly, standardized strategies promoting behavioral change and aiming to improve motivation could also be beneficial. While some general strategies such as motivational interviewing (Rollnick & Miller, 1995) are currently employed by many municipalities, standardized approaches tailored specifically to the needs of young adults with NDCs aiming to enable the transition to adulthood appear lacking in housing support. Existing approaches to facilitate the transition to adulthood for young people with NDCs typically focus on some common elements, including individualized goals, information/education, and self-determination (Jonsson et al., 2019; Organization for Autism Research, 2021). Evaluation of the feasibility, acceptability, and effectiveness of using such strategies in a structured way is an important next step. It is equally important to further investigate to what extent reasonable accommodations of the environment and greater awareness of neurodiversity in the society at large (e.g., work, higher education, and healthcare) can mitigate some of the support needs experienced by young adults with NDCs (Anderson et al., 2018; Bölte et al., 2021).

Future research should also consider the perspectives of other stakeholders, including service users, relatives, management representatives, and private service providers. The perspectives of young adults with NDCs themselves are particularly critical given that housing support is deemed a collaborative effort (van Schalkwyk & Dewinter, 2020), but the importance of collaboration with the family was also mentioned. Important questions include what difficulties the service users and their parents see in collaboration with support workers, and what they perceive as the main goals of the support. Understanding these perspectives can help identify when the objectives of the service provider and the young adults and their families do not fully align (Sosnowy et al., 2018).

The wide range of competencies drawn from many professional backgrounds (e.g., psychiatry, social work, psychology, pedagogy, and occupational therapy) needed to design and deliver high-quality housing support might be difficult to assemble in each municipality, especially the smaller ones. Therefore, the implementation of user-friendly guidelines may be particularly important to translate best practice and evidence into a sustainable and flexible service. Government agencies provide some guidance, including educational requirements for support workers, ICF use, and overarching support service principles (Swedish National Board of Health and Welfare, 2012, 2021a, 2022). Such guidance, if complemented by training for the support workers based on the available research on life transitions for people with NDCs, could potentially help inform support services that are tailored for the target group. Staff training might ideally be practically oriented hands-on instructions, focusing on neurodiversity in young people and how to organize and individualize the support based on such individual differences. Evaluations of both intended and unintended effects will be an important part of the process of developing and implementing such guidelines.

### Limitations

The results should be viewed in light of some limitations. Firstly, as mentioned above, the study was underpinned by the perspective of support workers only. Secondly, it is possible that the descriptions partly reflect a somewhat broader population than young adults with NDCs, given many teams provided support to a larger group of service users in terms of age and impairment. To counteract this, the participants were reminded to focus on young clients with ADHD and/or autism during the interview. While there is a range of other NDCs, these two are by far the most well-known and frequently occurring conditions in the context of housing support. However, the specific focus on ADHD and autism may have led the participants to neglect information about some other NDCs or co-occurrence with other conditions. Thirdly, given the relatively small number of participants representing this diverse support service, in combination with the heterogeneity of support needs, functioning, and abilities of the target group, it is unlikely that the present study captured all aspects of current practice. Fourthly, levels of pre-understanding about a topic can influence researchers preconceived notions, assumptions, and understanding (Graneheim et al., 2017). The interviews were performed by the first author, who had seven years of experience in delivering housing support to young adults with NDCs. If, for instance, the interviewer and the participants have the same presumptions and ‘blind spots’, there might be a risk that some relevant aspects may be overlooked. On the other hand, some level of understanding of a research topic can also be helpful in identifying meaning units (Krippendorff, 2019). In the present study, the authorship team had varying levels of familiarity with the service, enabling a balanced portrayal of the findings. The authors also had previous experience in research on NDCs and evaluation of complex interventions, which to some extent may have guided the conclusions. Fifthly, that interviews were conducted by telephone, which may have prevented the interviewer from picking up subtle aspects of the communication (e.g., body language). Some interviews were conducted in groups, which may have inhibited some participants, but could also potentially have enriched the discussions. Sixthly, the information we obtained about each participant was limited to some basic characteristics. It should be noted, however, that we were not primarily interested in the participating support worker’s specific experiences. Conversely, the participants were free to discuss the questions with their team ahead of the interview, to tap the experiences of a broader group of support workers. Finally, the transferability of the results may partly be restricted to support system similar to the Swedish one. In addition, young adults who receive housing support primarily live independently, suggesting that the results might not be transferable to young adults with more extensive support needs. On the other hand, we believe that the overall questions raised by the results have broad relevance for how support should be provided for this specific target group.

## Conclusion

While the support workers interviewed in this study conveyed that they had a strong commitment and many of the professional and interpersonal skills required for this work, there still seems to be a significant research-to-practice gap. There is a need for greater awareness of the specific support needs of young adults with NDC among both support workers and management. To this end, the perspectives of those with lived experience should be incorporated into the decision-making process at an organizational level. While more detailed guidance for support workers and management clearly is needed, it is also important to allow for flexibility in adapting to the service users’ diverse and shifting needs throughout their transition to adulthood.
